# 4478 Not just GLUT1: genome sequencing reveals genetic heterogeneity in Doose syndrome

**DOI:** 10.1017/cts.2020.84

**Published:** 2020-07-29

**Authors:** Jeffrey Dennis Calhoun, Jonathan Gunti, Katie Angione, Elizabeth Geiger, Krista Eschbach, Garnett Smith, Charuta Joshi, Tamim Shaikh, Scott Demarest, Gemma Carvill

**Affiliations:** 1 Northwestern University

## Abstract

OBJECTIVES/GOALS: Epilepsy with myoclonic-atonic seizures (EMAS) is a childhood onset epilepsy disorder characterized by seizures with sudden loss of posture, or drop seizures. Our objective was to use short-read genome sequencing in 40 EMAS trios to better understand variants contributing to the development of EMAS. METHODS/STUDY POPULATION: Eligibility for the cohort included a potential diagnosis of EMAS by child neurology faculty at Children’s Hospital Colorado. Exclusion criteria included lack of drop seizures upon chart review or structural abnormality on MRI. Some individuals had prior genetic testing and priority for genome sequencing was given to individuals without clear genetic diagnosis based on previous testing. We analyzed single nucleotide variants (SNVs), small insertions and deletions (INDELs), and larger structural variants (SVs) from trio genomes and determined those that were likely contributory based on standardized American College of Medical Genetics (ACMG) criteria. RESULTS/ANTICIPATED RESULTS: Our initial analysis focused on variants in coding regions of known epilepsy-associated genes. We identified pathogenic or likely pathogenic variants in 6 different individuals involving 6 unique genes. Of these, 5 are *de novo* SNVs or INDELs and 1 is a *de novo* SV. One of these involve a *de novo* heterozygous variant in an X-linked gene (*ARHGEF9*) in a female individual. We hypothesize the skewed X-inactivation may result in primarily expression of the pathogenic variant. We anticipate identifying additional candidate variants in coding regions of genes previously not associated with EMAS or pediatric epilepsies as well as in noncoding regions of the genome. DISCUSSION/SIGNIFICANCE OF IMPACT: Despite the genetic heterogeneity of EMAS, our initial analysis identified *de novo* pathogenic or likely pathogenic variants in 15% (6/40) of our cohort. As the cost continues to decline, short read genome sequencing represents a promising diagnostic tool for EMAS and other pediatric onset epilepsy syndromes. CONFLICT OF INTEREST DESCRIPTION: The authors have no conflicts of interest to disclose. SD has consulted for Upsher-Smith, Biomarin and Neurogene on an unrelated subject matter. GLC holds a research collaborative grant with Stoke therapeutics on unrelated subject matter.

